# Lupus Pernio in Chronic Sarcoidosis

**DOI:** 10.7759/cureus.18331

**Published:** 2021-09-27

**Authors:** Kaba Condé, Carlos Othon Guelngar, Awada Mohamed, Emmanuel Adjibaye, Fodé Abass Cissé

**Affiliations:** 1 Department of Rheumatology, Ignace Deen National Hospital, University of Conkary, Conakry, GIN; 2 Department of Neurology, Ignace Deen National Hospital, University of Conkary, Conakry, GIN; 3 Department of Infectious Disease, Reference Hospital, N'Djamena, TCD

**Keywords:** guinea, conakry, lupus pernio, chronic sarcoidosis, dactylitis

## Abstract

Lupus pernio (LP) is characterized by the association between insidious purpuric or purplish blue lesions localized in the nose, cheeks, lips, and ears and swelling of the fingers and toes. We report a case of chronic sarcoidosis with lupus pernio in a 34-year-old male. The diagnosis of sarcoidosis was made on the basis of clinical data and imaging results and confirmed by skin biopsy, which showed numerous epithelioid granulomas surrounded by a non-caseous inflammatory crown. Treatment with prednisolone was started. It is important to make an early diagnosis to avoid a delay in treatment and worsening of the functional and psychological prognosis.

## Introduction

Sarcoidosis is a chronic multisystem inflammatory granulomatosis of unknown origin. It can affect the pulmonary, skin, gastrointestinal, cardiac, musculoskeletal, endocrine, or central nervous system [1]. Lupus pernio (LP) was first described by Besnier in 1989 [2] and is considered as the most characteristic skin lesion of chronic sarcoidosis [3,4]. Lupus pernio is characterized by the association between insidious purpuric or purplish blue lesions localized in the nose, cheeks, lips, and ears and swelling of the fingers and toes [2,3]. It is present in 50% of patients with pulmonary sarcoidosis [5]. LP most often affects women aged 45-65 years and people with black skin; it can cause significant cosmetic damage [3,5]. We report a case of LP in chronic sarcoidosis in a 34-year-old male.

## Case presentation

Patient consent has been obtained for the publication of the case and associated images.

**Figure 1:**
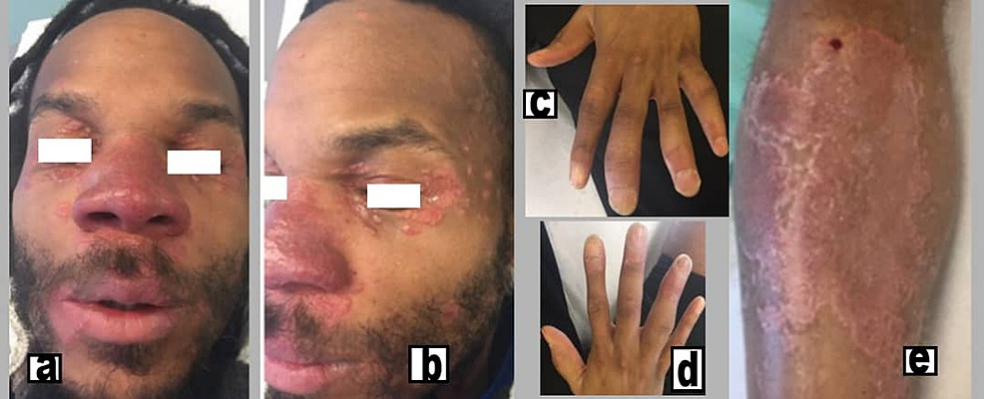
Photograph of the face, hands, and right leg. (a,b,e) Photograph of indurated, purplish plaques on the eyelids, nose, cheeks, and ears and on the inner side of the right leg. (c,d) Photograph of hands showing dactylitis of the proximal and distal interphalangeal joints.

A 34-year-old male patient with no particular history was seen in consultation for swelling of the face with deformation and the presence of a nodule in the nose, cheeks, and ears accompanied by purpuric purplish lesions on the right leg consistent with lupus pernio (Figure 1) slowly evolving over the last eight years. There was no notion of fever, nor of respiratory, cardiovascular, or arthralgic symptoms. The diagnosis of sarcoidosis was made on the basis of epidemiological arguments, clinical data, and imaging results and confirmed by skin biopsy, which showed numerous epithelioid granulomas surrounded by a non-caseous inflammatory crown. In May 2016, the patient developed chronic polysynovial arthritis progressing with relapses interspersed with remissions, deforming bilaterally and symmetrically, affecting the metacarpophalangeal (MCP), proximal interphalangeal (PIP), distal interphalangeal (DIP), and the first metatarsophalangeal (MTP) joints. On physical examination, the patient was in good general condition. There were pain and swelling (swelling and dactylitis appearances) of the MCP, PIP, DIP, and MTP2-3 bilaterally (Figures 1c, 1d). Dermatologically, there were purplish, painless, hardened plaques located on the nose, cheeks, and lips (Figures 1a, 1b) and the medial surface of the right leg (Figure 1e). Cardiopulmonary, abdominal, and ophthalmologic examinations were normal.

**Figure 2:**
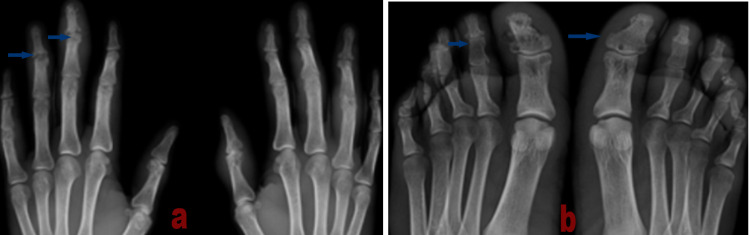
Radiograph of the hands and feet. (a) Radiograph of the hands showing multiple geodes and osteolysis at the proximal and distal interphalangeal joints. (b) X-ray of the feet showing subchondral geodes facing the interphalangeal space of the bilateral hallux and osteolysis of the fourth right ray.

Biological evaluation showed an inflammatory syndrome with a sedimentation rate of 104 mm/h and a C-reactive protein concentration of 10.1 mg/L, associated with polyclonal hypergammaglobulinemia. The angiotensin-converting enzyme level elevated to 64 IU/L (normal value: 20-60 IU/L), and the calcium level increased to 3 mmol/L (normal value: 2.2-2.6 mmol/L), suggesting hypercalcemia. There was also an increase in immunoglobulin G4 (IgG4) to 298 mg/dL. Renal and hepatic functions were normal. Antinuclear antibodies, rheumatoid factor, and anti-cyclic citrullinated peptide antibodies were negative. A pulmonary function test found a slight restrictive deficit with a significant decrease in diffusion. X-ray of the hands and feet (Figures 2a, 2b) showed destructive arthropathy with multiple subchondral geodes and bone erosion, with acro-osteolysis of the MCP, PIP, DIP, and MTP1-2.

**Figure 3:**
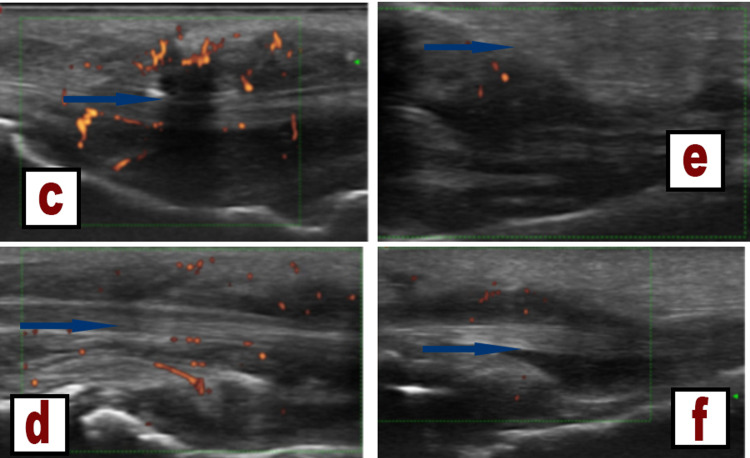
Ultrasonography of the hands and feet. (c) Ultrasonography of the hands showing grade 2 inflammatory tenosynovitis in the right flexor digitorum longus. (d) Ultrasound of the hands showing a major dactylitis of the fourth finger on the left. (e) Ultrasound of the feet showing grade 2 inflammatory tenosynovitis of the flexor 1 of the toes. (f) Ultrasound of the feet showing grade 2 inflammatory tenosynovitis of the flexor 2 of the toes.

**Figure 4:**
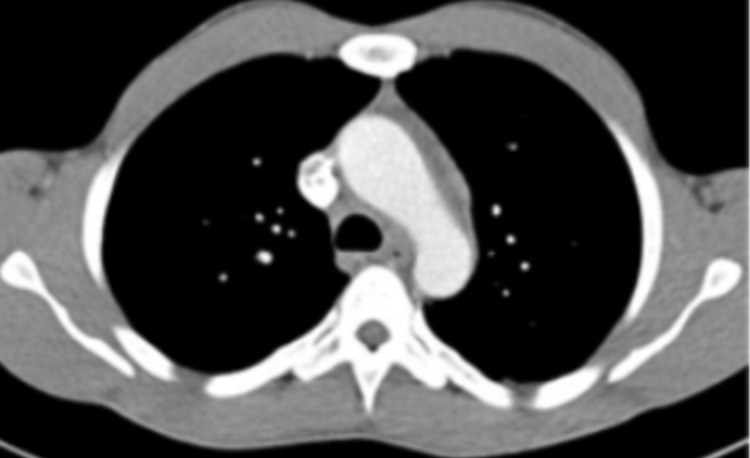
Chest CT scan. Chest CT scan showing bilateral diffuse subpleural and perifissural micronodules.

Ultrasound of the hands and feet (Figures 3c-3f) showed inflammatory synovitis, inflammatory tenosynovitis, and major dactylitis in the MCP, PIP, DIP, and MTP1-2 bilaterally. Chest CT scan (Figure 4) showed several bilateral subpleural and diffuse perifissural micronodules predominantly on the right. We, therefore, retained the diagnosis of chronic sarcoidosis with lupus pernio. Medrol treatment has been initiated.

## Discussion

LP was first described by Besnier in 1889 [2] and is considered as the most characteristic skin lesion in chronic sarcoidosis [3,4]. The disease frequency varies from 2.7% to 11.8% during sarcoidosis [6,7], and it is more common in women of African and Asian origin [5,6]. The incidence of chronic arthritis associated with sarcoidosis is 1%-4% [8]. We report the case of a 34-year-old male patient. Although the most characteristic expression of LP is extensive involvement of the skin of the nose, it may also be presented by plaques on the cheeks, ears, and forehead or small nodules at the tip and base of the nose, DIP, and toes [3]. However, our patient had skin involvement all over his face, hands, feet, and right leg. LP is often associated with upper respiratory tract damage [8]. The etiology of sarcoidosis is unknown. About 50%-75% of patients with sarcoidosis present with decreased T lymphocyte function and increased B lymphocyte activity (high levels of IgA and IgG) [9], as observed in our patient. The diagnosis of sarcoidosis is based on physical examination, chest X-ray, and serological tests such as angiotensin-converting enzyme, as it is elevated in 60% of cases of active sarcoidosis and hypercalcemia [10]. Biopsy of clinically suspicious lesions is the most reliable and accurate tool [11]. Many treatment options are available in the literature on the treatment of sarcoidosis, but few randomized trials are available dealing with the various treatment options for skin manifestations [11]. In all cases, LP requires treatment due to its disfiguring nature [11,12]. Despite the lack of evidence for the treatment of LP, topical corticosteroid therapy has shown favorable responses. Systemic corticosteroids are used for thick plaques unresponsive to topical corticosteroids and where intralesional corticosteroids would be impractical and painful [12,13]. Systemic glucocorticoids and intralesional therapy have been associated with excellent results and almost complete resolution [14]. Antimalarials, methotrexate, azathioprine, cyclophosphamide, thalidomide, and infliximab are used as corticosteroid spares [14]. Our patient was treated with Medrol 16 mg/day.

## Conclusions

Lupus pernio is specific to chronic sarcoidosis and is more common in populations of African and Asian origin. Although the prognosis is not life-threatening, patients with lupus pernio have an impaired quality of life, especially aesthetically. Early diagnosis is necessary to avoid a delay in treatment and a worsening prognosis.
